# Improved Genome Assembly and Annotation for the Rock Pigeon (*Columba livia*)

**DOI:** 10.1534/g3.117.300443

**Published:** 2018-03-08

**Authors:** Carson Holt, Michael Campbell, David A. Keays, Nathaniel Edelman, Aurélie Kapusta, Emily Maclary, Eric T. Domyan, Alexander Suh, Wesley C. Warren, Mark Yandell, M. Thomas P. Gilbert, Michael D. Shapiro

**Affiliations:** * Department of Human Genetics, University of Utah, Salt Lake City, UT, USA; † USTAR Center for Genetic Discovery, University of Utah, Salt Lake City, UT, USA; ‡ Research Institute of Molecular Pathology, Vienna, Austria; § Department of Biology, University of Utah, Salt Lake City, UT, USA; ** Department of Biology, Utah Valley University, Orem, UT, USA; †† Department of Evolutionary Biology (EBC), University of Uppsala, Uppsala, Sweden; ‡‡ Genome Institute at Washington University, St. Louis, MO, USA; §§ Natural History Museum of Denmark, University of Copenhagen, Copenhagen, Denmark; *** Norwegian University of Science and Technology, University Museum, 7491 Trondheim, Norway

**Keywords:** *Columba livia*, rock pigeon, HiRise assembly, MAKER annotation, Genome Report

## Abstract

The domestic rock pigeon (*Columba livia*) is among the most widely distributed and phenotypically diverse avian species. *C. livia* is broadly studied in ecology, genetics, physiology, behavior, and evolutionary biology, and has recently emerged as a model for understanding the molecular basis of anatomical diversity, the magnetic sense, and other key aspects of avian biology. Here we report an update to the *C. livia* genome reference assembly and gene annotation dataset. Greatly increased scaffold lengths in the updated reference assembly, along with an updated annotation set, provide improved tools for evolutionary and functional genetic studies of the pigeon, and for comparative avian genomics in general.

Intensive selective breeding of the domestic rock pigeon (*Columba livia*) has resulted in more than 350 breeds that display extreme differences in morphology and behavior (Levi 1986; Domyan and Shapiro 2017). The large phenotypic differences among different breeds make them a useful model for studying the genetic basis of radical phenotypic changes, which are more typically found among different species rather than within a single species.

In genetic and genomic studies of *C. livia*, linkage analysis is important for identifying genotypes associated with specific phenotypic traits of interest (Domyan and Shapiro 2017); however, short scaffold sizes in the Cliv_1.0 draft reference assembly (Shapiro *et al.* 2013) hinder computationally-based comparative analyses. Short scaffolds also make it more difficult to identify structural changes, such as large insertions or deletions, that are responsible for traits of interest (Domyan *et al.* 2014; Kronenberg *et al.* 2015).

Here we present the Cliv_2.1 reference assembly and an updated gene annotation set. The new assembly greatly improves scaffold length over the previous draft reference assembly, and updated gene annotations show improved concordance with both transcriptome and protein homology evidence.

## Materials & Methods

### Genome sequencing and assembly

Genomic DNA from a female Danish tumbler pigeon (full sibling of the male bird used for the original Cliv_1.0 assembly; Shapiro *et al.* 2013) was extracted from blood using a modified “salting out” protocol (Miller *et al.* 1988; modifications from http://www.protocol-online.org/prot/Protocols/Extraction-of-genomic-DNA-from-whole-blood-3171.html, accessed February 06, 2018). Blood was frozen immediately after collection and stored at -80°, and purified DNA was resuspended in 10 mM Tris-HCl. The sample went through 2 freeze-thaw cycles before being used to construct the libraries described below.

Extracted DNA was used to produce long-range sequencing libraries using the “Chicago” method (Putnam *et al.* 2016) by Dovetail Genomics (Santa Cruz, CA). Two Chicago libraries were prepared and sequenced on the Illumina HiSeq platform to a final physical coverage (1-50 kb pairs) of 390x.

Scaffolding was performed by Dovetail Genomics using HiRise assembly software and the Cliv_1.0 assembly as input. Briefly, Chicago reads were aligned to the input assembly to identify and mask repetitive regions, and then a likelihood model was applied to identify mis-joins and score prospective joins for scaffolding. The final assembly was then filtered for length and gaps according to NCBI submission specifications.

### Custom repeat library

A repeat library for *C. livia* was built by combining libraries from existing avian genome assemblies (Zhang *et al.* 2014a) together with repeats identified *de novo* for the Cliv_2.1 assembly. *De novo* repeat identification was performed using RepeatScout (Price *et al.* 2005) with default parameters (>3 copies) to generate consensus repeat sequences. Identified repeats with greater than 90% sequence identity and a minimum overlap of 100 bp were assembled using Sequencher (Gene Codes Corporation, Ann Arbor, MI). Repeats were classified into transposable element (TE) families using multiple lines of evidence, including homology to known elements, presence of terminal inverted repeats (TIRs), and detection of target site duplications (TSDs). Homology-based evidence was obtained using RepeatMasker (Smit *et al.* 1996), as well as the homology module of the TE classifying tool RepClass (Feschotte *et al.* 2009). RepClass was also used to identify signatures of transposable elements (TIRs, TSDs). We then eliminated non-TE repeats (simple repeats or gene families) using custom Perl scripts (available at https://github.com/4ureliek/ReannTE).

Our custom repeat analysis used the script ReannTE_FilterLow.pl to label consensus sequences as simple repeats or low complexity repeats if 80% of their length could be annotated as such by RepeatMasker (the library was masked with the -noint option). Next, we used the ReannTE_Filter-mRNA.pl script to compare consensus sequences to RefSeq (Pruitt *et al.* 2007) mRNAs (as of March 7^th^ 2016) with TBLASTX (Altschul *et al.* 1990). Sequences were eliminated from the library when: (i) the e-value of the hit was lower than 1E-10; (ii) the consensus sequence was not annotated as a TE; and (iii) the hit was not annotated as a transposase or an unclassified protein. The script ReannTE_MergeFasta.pl was then used to merge our library with a library combining RepeatModeler (Smit and Hubley 2008) outputs from 45 bird species (Kapusta *et al.* 2017) and complemented with additional avian TE annotations (International Chicken Genome Sequencing Consortium 2004; Warren *et al.* 2010; Bao *et al.* 2015). Merged outputs were manually inspected to remove redundancy, and all DNA and RTE class transposable elements were removed and replaced with manually curated consensus sequences, which were either newly (DNA elements) or previously generated (RTEs) (Suh *et al.* 2016).

### Repeat landscape

We used RepeatMasker software v4.0.7 (Smit *et al.* 2015) and our custom library to annotate the repeats in Cliv_2.1. RepeatMasker was run with the NCBI/RMBLAST v2.6.0+ search engine (-e ncbi), the sensitive (-s) option, the -a option in order to obtain the alignment file, and without RepeatMasker default libraries. We then used the parseRM.pl script v5.7 (available at https://github.com/4ureliek/Parsing-RepeatMasker-Outputs; Kapusta *et al.* 2017), on the alignment files from RepeatMasker, with the -l option and a substitution rate of 0.002068 substitutions per site per million years (Zhang *et al.* 2014b). The script collects the percentage of divergence from the consensus for each TE fragment, after correction for higher mutation rate at CpG sites and the Kimura 2-Parameter divergence metric (provided in the alignment files from RepeatMasker). The percentage of divergence to the consensus is a proxy for age (the older the TE invasion, the more mutations will accumulate in TE fragments), to which the script applies the substitution rate in order to split TE fragments into bins of 1 My.

### Transcriptomics

RNA was extracted from adult tissues (brain, retina, subepidermis, cochlear duct, spleen, olfactory epithelium) of the racing homer breed, and one whole embryo each of a racing homer and a parlor roller (approximately embryonic stage 25; Hamburger and Hamilton 1951). RNA-seq libararies were prepared and sequenced using 100-bp paired-end sequencing on the Illumina HiSeq 2000 platform at the Research Institute of Molecular Pathology, Vienna (adult tissues), and the Genome Institute at Washington University, St. Louis (embryos). RNA-seq data generated for the Cliv_1.0 annotation were also downloaded from the NCBI public repository for *de novo* re-assembly. Accession numbers for these public data are SRR521357 (Danish tumbler heart), SRR521358 (Danish tumbler liver), SRR521359 (Oriental frill heart), SRR521360 (Oriental frill liver), SRR521361 (Racing homer heart), and SRR521362 (Racing homer liver).

Each FASTQ file was processed with FastQC (http://www.bioinformatics.babraham.ac.uk/projects/fastqc/) to assess quality. When FastQC reported overrepresentation of Illumina adapter sequences, we trimmed these sequences with fastx_clipper from the FASTX-Toolkit (http://hannonlab.cshl.edu/fastx_toolkit/). We used FASTX-Toolkit for two additional functions: runs of low quality bases at the start of reads were trimmed with fastx_trimmer when necessary (quality cutoff of -Q 33), and reads were then trimmed with fastq_quality_trimmer (-Q 33). Finally, each pair of sequence files was assembled with Trinity (Grabherr *et al.* 2011) version r20131110 using the –jaccard_clip option.

### Genome annotation

The pre-existing reference Gnomon (Souvorov *et al.* 2010) derived gene models for the Cliv_1.0 assembly (GCA_000337935.1) were mapped onto the updated Cliv_2.1 reference assembly using direct alignment of transcript FASTA entries. This was done using the alignment workflow of the genome annotation pipeline MAKER (Cantarel *et al.* 2008; Holt and Yandell 2011), which first seeds alignments using BLASTN (Altschul *et al.* 1990) and then polishes the alignments around splice sites using Exonerate (Slater and Birney 2005). Results were then filtered to remove alignments that had an overall match of less than 90% of the original model (match is calculated as percent identity multiplied by percent end-to-end coverage).

For final annotation, MAKER was allowed to identify *de novo* gene models that did not overlap the aligned Gnomon models. Protein evidence sets used by MAKER included annotated proteins from *Pterocles gutturalis* (yellow-throated sandgrouse; Zhang *et al.* 2014a) and *Gallus gallus* (chicken; International Chicken Genome Sequencing Consortium 2004) together with all proteins from the UniProt/Swiss-Prot database (Bairoch and Apweiler 2000; UniProt Consortium 2007). The transcriptome evidence sets for MAKER included Trinity mRNA-seq assemblies from multiple *C. livia* breeds and tissues (methods for transcriptome assembly are described above). Gene predictions were produced within MAKER by Augustus (Stanke and Waack 2003; Stanke *et al.* 2008). Augustus was trained using 1000 Cliv_1.0 Gnomon gene models that were split using the randomSplit.pl script into sets for training and evaluation. We followed a semi-automatic training protocol (https://vcru.wisc.edu/simonlab/bioinformatics/programs/augustus/docs/tutorial2015/training.html, accessed February 9, 2018). Repetitive elements in the genome were identified using the custom repeat library described above.

### Linkage map construction and anchoring to current assembly

Genotyping-by-sequencing (GBS) data were generated, trimmed, and filtered as previously described (Domyan *et al.* 2016). Reads were mapped to the Cliv_2.1 assembly using Bowtie2 (Langmead and Salzberg 2012). Genotypes were called using Stacks v1.46 (Catchen *et al.* 2011), with a minimum read-depth cutoff of 10. Thresholds for automatic corrections were set using the parameters –min_hom_sequations 10, –min_het_seqs 0.01, –max_het_seqs 0.15. Sequencing coverage and genotyping rate varied between individuals, and birds with genotyping rates in the bottom 25% were excluded from map assembly.

Genetic map construction was performed using R/qtl v1.41-6 (www.rqtl.org; Broman *et al.* 2003). For autosomal markers, markers showing segregation distortion (Chi-square, *P* < 0.01) were eliminated. Sex-linked scaffolds were assembled and ordered separately, due to differences in segregation pattern for the Z-chromosome. Z-linked scaffolds were identified by assessing sequence similarity and gene content between pigeon scaffolds and the Z-chromosome of the annotated chicken genome (Ensembl Gallus_gallus-5.0).

Pairwise recombination fractions were calculated in R/qtl for all autosomal and Z-linked markers. Missing data were imputed using “fill.geno” with the method “no_dbl_XO”. Duplicate markers were identified and removed. Within individual scaffolds, R/qtl functions “droponemarker” and “calc.errorlod” were used to assess genotyping error. Markers were removed if dropping the marker led to an increased LOD score, or if removing a non-terminal marker led to a decrease in length of >10 cM that was not supported by physical distance. Individual genotypes were removed if they showed error LOD scores >5 (Lincoln and Lander 1992). Linkage groups were assembled from 2960 autosomal markers and 232 Z-linked markers using the parameters (max.rf 0.1, min.lod 6). In the rare instance that single scaffolds were split into multiple linkage groups, linkage groups were merged if supported by recombination fraction data; these instances typically reflected large physical gaps between markers on a single scaffold. Scaffolds in the same linkage group were manually ordered based on calculated recombination fractions and LOD scores.

To compare the linkage map to the original genome assembly (Cliv_1.0), each 90-bp locus containing a genetic marker was parsed from the Stacks output file “catalogXXX_tags.tsv” and queried to the Cliv_1.0 assembly using BLASTN (v2.6.0+) with the parameters –max_target_sequations 1 –max_hsps 1. 3,175 of the 3,192 loci (99.47%) from the new assembly had a BLAST hit with an E-value < 4e-24 and were retained.

### Assembly comparisons

FASTA files from the Cliv_2.1 and colLiv2 (Damas *et al.* 2017) genome assemblies were hard masked using NCBI WindowMasker (Morgulis *et al.* 2006) and genome-wide alignments were calculated with LAST (Kielbasa *et al.* 2011). From these alignments, a genome-scale dotplot indicating syntenic regions was generated using SynMap (Lyons and Freeling 2008; Lyons *et al.* 2008).

The colLiv2 assembly is currently unannotated. Therefore, to compare gene content between assemblies, we estimated the number of annotated Cliv_2.1 genes absent from colLiv2 based on gene coordinates. Based on the length of LAST alignments, we calculated the percent of each Cliv_2.1 scaffold aligning to colLiv2. Scaffolds were divided into four groups based on alignments: Cliv_2.1 scaffolds that did not align to colLiv2, Cliv_2.1 scaffolds where LAST alignments to colLiv2 covered less than 50% of the total scaffold length, Cliv_2.1 scaffolds where LAST alignments to colLiv2 covered between 50% and 75% of the total scaffold length, and Cliv_2.1 scaffolds where LAST alignments to colLiv2 covered 75% or more of the total scaffold length. For each of these groups, the number of scaffolds containing genes was quantified. Many of these scaffolds are small, and some may be partially or completely missing from the alignment due to masking of repetitive elements. If annotated gene coordinates from Cliv_2.1 scaffolds fell partially or entirely within a region aligned to colLiv2, these genes were considered “present” in colLiv2. Thus, the number of genes marked as “absent” in colLiv2 might be a conservative estimate.

To compare the linkage map to colLiv2, each 90-bp locus containing a genetic marker was parsed from the Stacks output file “catalogXXX_tags.tsv” and queried to the colLiv2 assembly using BLASTN (v2.6.0+) with the parameters –max_target_seqs 1 –max hsps 1.

### Data availability

This Whole Genome Shotgun project has been deposited at DDBJ/ENA/GenBank under the accession AKCR00000000. The version described in this paper is version AKCR02000000. The Cliv_2.1 assembly, annotation, and associated data are available at ftp://ftp.ncbi.nlm.nih.gov/genomes/all/GCA/000/337/935/GCA_000337935.2_Cliv_2.1.

RNA-seq data are deposited in the SRA database with the BioSample accession numbers SAMN07417936-SAMN07417943, and sequence accessions SRR5878849-SRR5878856. Assembly and RNA-seq data are publicly available in NCBI databases under BioProject PRJNA167554. File S1 contains Tables S1–S7. File S2 and File S3 contain recombination fraction data used to construct Figures 5a and 5b, respectively.

## Results and Discussion

### Genome assembly

**Figure 1:**
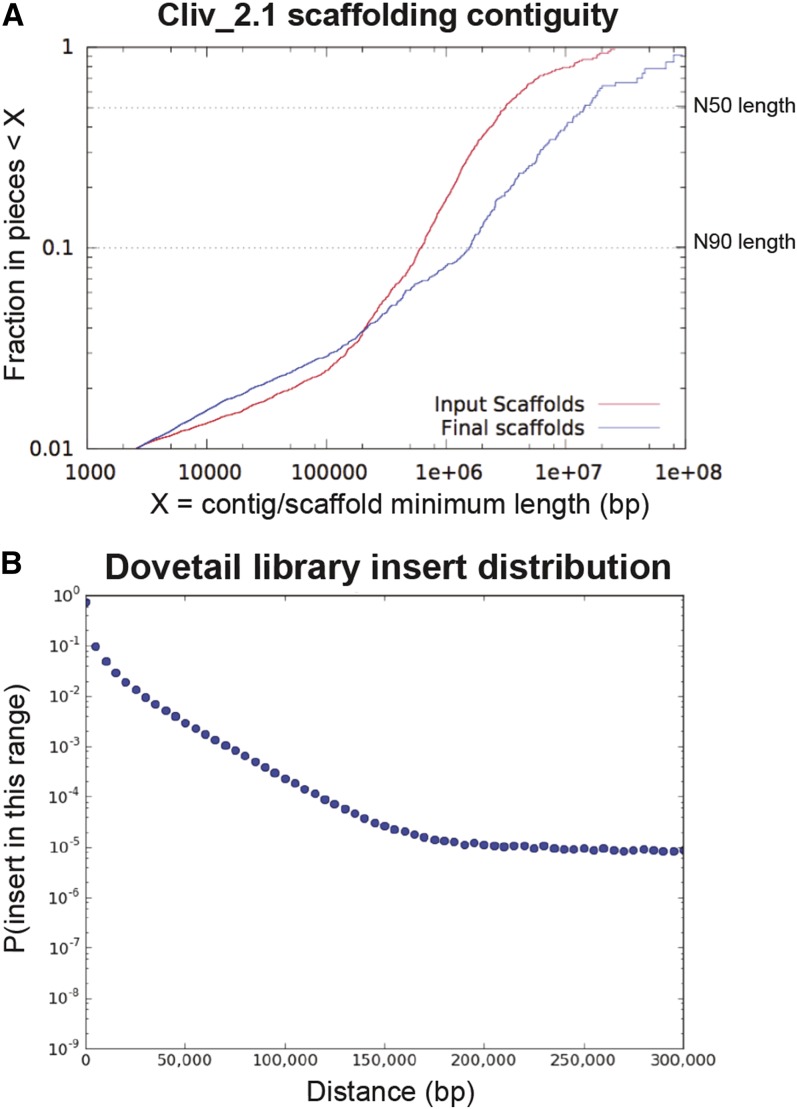
Assembly scaffolding contiguity and scaffolding library insert size distributions. (a) Scaffolding comparison between Cliv_1.0 (input scaffolds) and Cliv_2.1 (final scaffolds) assemblies. (b) Distribution of Dovetail Genomics “Chicago” library inserts.

The final Cliv_2.1 reference assembly is 1,108,534,737 base pairs in length and consists of 15,057 scaffolds (Table 1). A total of 1,015 scaffolds contain a gene annotation. Completion analysis of the assembly using BUSCO v2 and the odb9 Vertebrata ortholog dataset (Simão *et al.* 2015) suggests that Cliv_2.1 is 72.9 (assembly) to 86.2% (annotation) complete. These statistics are nearly identical to the Cliv_1.0 assembly estimate of 72.3–86.4% (Table 2); therefore, we found no significant changes in completeness between the two assemblies. Because the Chicago libraries and HiRise assembly were designed to improve scaffolding of the original assembly, not to fill gaps, we did not expect substantial improvement to assembly completeness in Cliv_2.1. Instead, the major improvement to the Cliv_2.1 assembly is a substantial increase in scaffold length (Figure 1a). The N50 scaffold length for Cliv_2.1 increased to 14.3 megabases, compared to 3.15 megabases for Cliv_1.0, a greater than fourfold increase.

**Table 1:** Assembly statistics for Cliv_2.1

Parameter	Value
Estimated Physical Coverage	389.7x
Total Length	1,108,534,737 bp
Total scaffolds	15,057
Total scaffolds >1kb	4,062
Total scaffolds >10kb	848

**Table 2:** Assembly version comparison

	Cliv_1.0	Cliv_2.1
Total Length	1110.8 Mb	1110.9 Mb
N50 Length	3.15 Mb and 82 scaffolds	14.3 Mb and 17 scaffolds
N90 Length	0.618 Mb and 394 scaffolds	1.56 Mb and 113 scaffolds
Completeness Estimate	72.3–86.4%	72.9–86.2%

The new assembly joins scaffolds that, based on linkage mapping evidence (Domyan *et al.* 2016), we knew were physically adjacent but were still separated in Cliv_1.0 (see Table S1 in File S1 for full catalog of positions of the original assembly in the new assembly, and Table S2 in File S1 for full catalog of breaks in the original assembly to form the new assembly). For example, we previously determined that Cliv_1.0 Scaffolds 70 and 95 were joined based on genetic linkage data from a laboratory cross (Domyan *et al.* 2016). These two sequences are now joined into a single scaffold in the Cliv_2.1 assembly (see Table S6 in File S1 for positions of genetic markers in Cliv_1.0 and Cliv_2.1). At least one gene model (RefSeq LOC102093126), which was previously split across two contigs, has now been unified into a single model on a single scaffold.

### Repeat landscape

**Figure 2:**
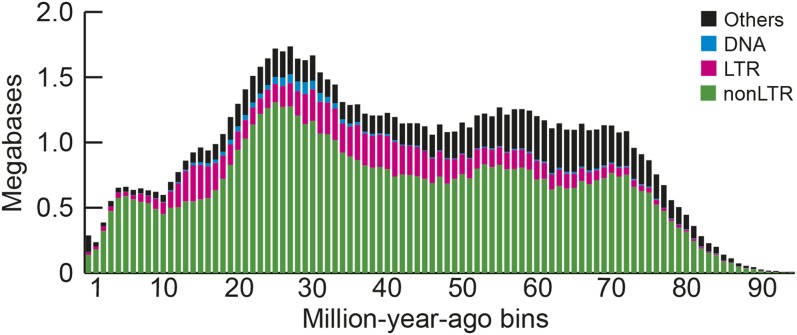
Temporal landscape of transposable elements. The amounts of DNA in each TE class were split into bins of 1 My, shown on the x axis (see Methods). We note that the lower detection of older elements (right of the graph) comes from a combination of lack of detection and TE removal, and that the amount of DNA corresponding to recent elements may be underestimated (recent copies are often collapsed in assemblies). The “Others” category primarily includes unclassified repeats.

Using our custom library, we identified 8.04% (89.1 Mb; Table S3 in File S1) of the genome assembly as repeats, which is slightly higher than the previously published estimates of 7.25% (Zhang *et al.* 2014b) and 7.83% (Kapusta and Suh 2017). To illustrate the temporal dynamics of TE accumulation (see Methods), we split the amount of DNA of each TE class by bins of 1 million years (My) (Figure 2). This landscape shows that TE accumulation has been consistent throughout time, with some potentially recently active elements. This includes CR1 LINEs (part of the non-LTR fraction), which are presumed to be inactive in most birds (Kapusta and Suh 2017), but comprise over 0.1 Mb of CR1 copies in the youngest bin (0-1 My) in the Cliv_2.1 assembly (Table S4 in File S1).

### Transcriptome assemblies

A total of 1,936,543 transcripts were assembled from the 14 RNA-seq data sets. Numbers of assembled transcripts from each tissue are listed in Table 3. BUSCO analysis indicated 85.6% completeness of the union of transcriptome assemblies compared to the Vertebrata ortholog set.

**Table 3:** Transcriptome assembly summary

SRA accession	Tissue	Breed	# assembled transcripts
SRR521357	Heart	Danish tumbler	79,473
SRR521358	Liver	Danish tumbler	35,691
SRR521359	Heart	Oriental frill	71,078
SRR521360	Liver	Oriental frill	74,180
SRR521361	Heart	racing homer	80,034
SRR521362	Liver	racing homer	80,642
SRR5878849	Embryo	racing homer	208,682
SRR5878850	Embryo	parlor roller	344,735
SRR5878851	Spleen	racing homer	156,415
SRR5878852	Olfactory epithelium	racing homer	112,632
SRR5878853	Subepidermis	racing homer	185,484
SRR5878854	Cochlear duct	racing homer	189,438
SRR5878855	Brain	racing homer	131,999
SRR5878856	Retina	racing homer	186,060

### Annotation

The updated annotation set contains 15,392 gene models encoding 18,966 transcripts (Table 4). This represents a minor update of the reference annotation set as 94.7% of previous models were mapped forward nearly unmodified (90% exact match for 14,898 out of 15,724 previous gene models) and 494 new gene models were added to the Cliv_2.1 annotation set (Table 5).

**Table 4:** Annotation statistics for Cliv_2.1

	Genes	Transcripts
Total	15,392	18,966
match*^a^*	14,898	18,472
new	494	494

a Count that match Cliv_1.0 annotations with a value of at least 90% (match is calculated as % identity multiplied by % end-to-end coverage).

**Table 5:** Annotation version comparison

	Cliv_1.0	Cliv_2.1
Total Gene Models	15,724	15,392
*coding*	15,022	14,683
*non-coding*	702	709
Total Transcripts	19,585	18,966
*coding*	18,569	18,148
*non-coding*	1016	818

**Figure 3:**
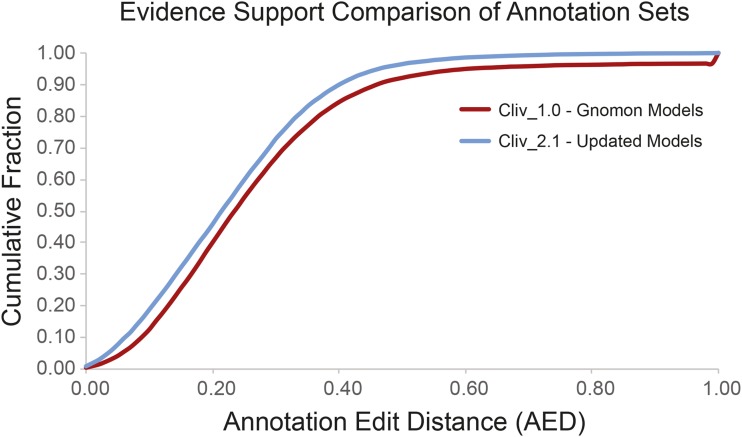
Evidence support comparison of annotation sets. Annotation edit distance (AED) support for gene models in Cliv_2.1 (blue line) is improved over Cliv_1.0 (NCBI Gnomon annotation, red line).

The updated annotation set shows a modest improvement in concordance with aligned evidence datasets from mRNA-seq and cross species protein homology evidence relative to the Cliv_1.0 set as measured by Annotation Edit Distance (AED; Eilbeck *et al.* 2009; Holt and Yandell 2011). As a result, transcript models in the Cliv_2.1 annotation tend to have lower AED values than the Cliv_1.0 set (Figure 3; the cumulative distribution function (CDF) curve is shifted to the left). Lower AED values indicate greater model concordance with aligned transcriptome and protein homology data. Furthermore, the Cliv_2.1 dataset displays greater transcript counts in every AED bin despite having slightly fewer transcripts overall compared to the Cliv_1.0 dataset (Table S5 in File S1). The higher bin counts indicate that lower AED values are not solely a result of removing unsupported models from the annotation set, but rather suggest that evidence concordance has improved overall.

### Linkage map

The linkage map consists of 3,192 markers assembled into 48 autosomal linkage groups and a single Z-chromosome linkage group (Table S6 in File S1). The map contains markers from 236 scaffolds. Together, these scaffolds encompass 1,048,536,443 bp (94.6%) of the Cliv_2.1 assembly, and include 13,026 of 15,392 (84.6%) annotated genes. Cliv_2.1 scaffolds are strongly supported by linkage data. For 235 out of 236 scaffolds included in the linkage map, all GBS markers mapped to that scaffold form a single contiguous block within one linkage group (only scaffold ScoHet5_252 was split between two linkage groups). Additionally, within-scaffold marker order was largely supported by calculated pairwise recombination fractions.

### Comparison with colLiv2 genome assembly

Recently, Damas *et al.* (2017) used computational methods and universal avian bacterial artificial chromosome (BAC) probes to achieve chromosome-level scaffolding of the pigeon genome using the Cliv_1.0 assembly as input material. This assembly, named colLiv2 (GenBank assembly accession GCA_001887795.1; 1,018,016,946 bp in length), is approximately 8% smaller than the Cliv_2.1 assembly.

**Figure 4:**
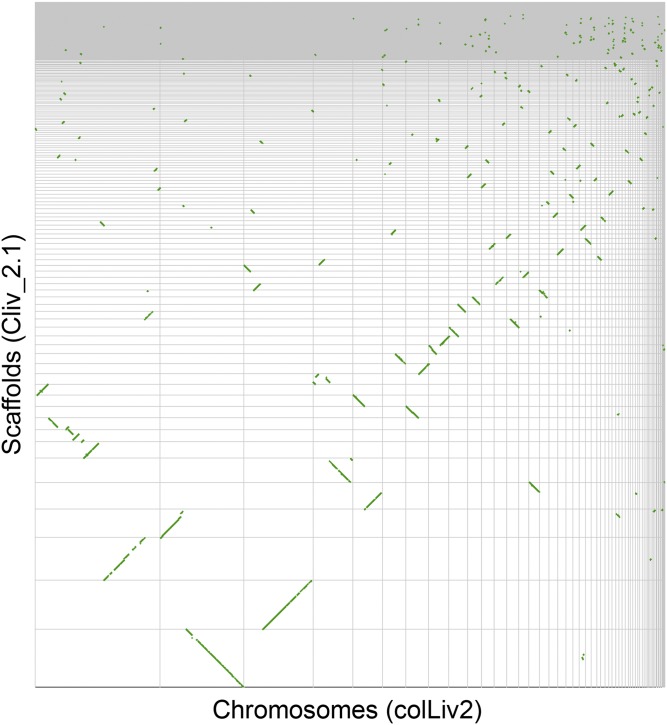
Dot plot of syntenic regions between the Cliv_2.1 and colLiv2 assemblies of the *C. livia* genome. Each segment of the X axis represents a single colLiv2 scaffold ordered from largest (left) to smallest (right), while each segment of the Y axis represents a scaffold of the Cliv_2.1 assembly, ordered from largest (bottom) to smallest (top). Green dots indicate aligned regions of synteny.

Based on genome-wide pairwise alignments using LAST (Figure 4) (Kielbasa *et al.* 2011), a substantial number of regions of Cliv_2.1 that do not align to colLiv2 genome contain both unique sequence and annotated genes. Based on gene coordinates, 1184 annotated Cliv_2.1 genes were absent from colLiv2 (Table 6).

**Table 6:** Summary of Cliv_2.1 alignment to colLiv2 chromosome-level scaffolds. Overall, colLiv2 appears to exclude 1,184, or approximately 7.7%, of the 15,392 annotated genes from the Cliv_2.1 assembly; this is consistent with the overall decrease in genome size

Cliv_2.1 scaffold representation	# of scaffolds	Scaffold length range	Scaffolds with genes	# of genes	Genes in LAST alignment to colLiv2	Genes missing from LAST alignment to colLiv2
Missing	14,189	200-393,647	147	164	NA	164
≤50% aligned	251	318-2,545,801	183	506	369	137
50–75% aligned	183	581-5,717,624	251	638	550	88
≥75% aligned	434	259-94,473,889	434	14,084	13,289	795

**Figure 5:**
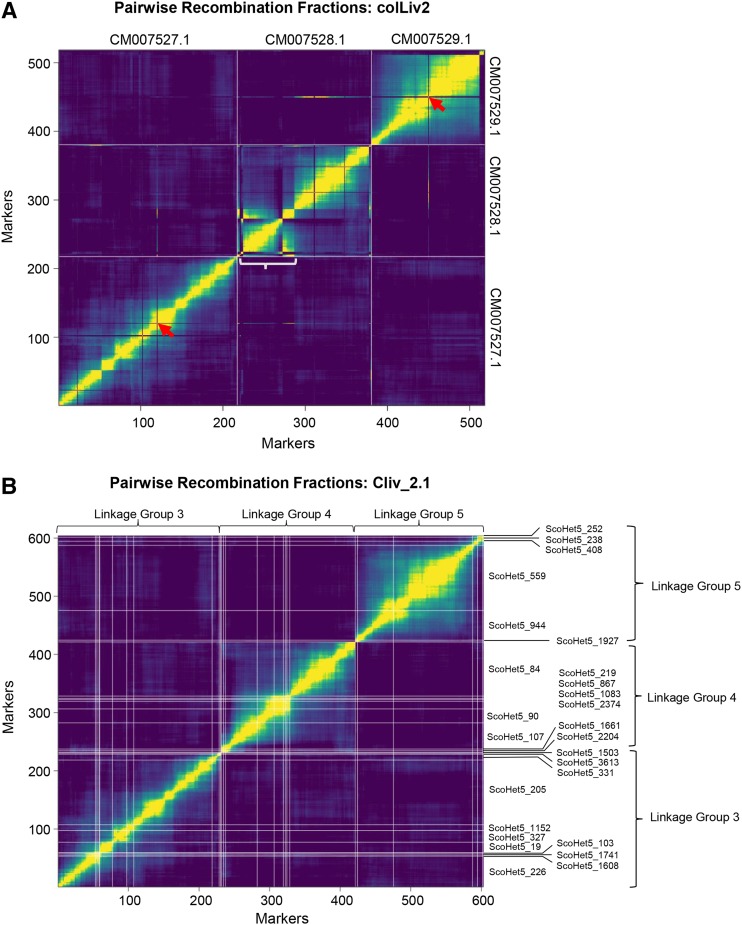
Correspondence between genotyping data and marker order in colLiv2 and Cliv_2.1 assemblies. (a) Representative plot of pairwise recombination fractions for GBS markers, ordered based on best alignment to colLiv2 assembly, for chromosomes CM007527.1, CM007528.1, and CM007529.1. X and Y axes show individual markers, ordered as they map to the colLiv2 chromosomes CM007527.1, CM007528.1, and CM007529.1. White lines mark the boundaries between chromosomes. Yellow indicates low pairwise recombination fraction (linked markers), while purple indicates high pairwise recombination fraction (unlinked markers). Red arrows highlight two markers, one mapped to chromosome CM007527.1 and one mapped to CM007529.1, for which recombination fractions suggest that these markers should instead be located on chromosome CM007528.1. A white bracket indicates a region on chromosome CM007528.1 where portions of the chromosome appear to be assembled in the wrong order. (b) Plot of pairwise recombination fractions for the Cliv_2.1 scaffolds that make up linkage groups 3, 4, and 5. In (a), colLiv2 CM007527.1 largely corresponds to linkage group 3, CM007528.1 to linkage group 4, and CM007529.1 to linkage group 5. White lines mark the boundaries between individual scaffolds, with scaffold IDs indicated on the right side.

Of the 3,192 GBS makers mapped to Cliv_2.1, 2,940 markers (92.1%) mapped to colLiv2 with an E-value <4e-24. Of the remaining markers, 7 mapped to colLiv2 with an E-value >4e-24, and 245 markers (7.67%) failed to map to colLiv2 entirely. We assessed the agreement between marker and linkage data by calculating pairwise recombination fractions for the 2940 markers, then plotted these recombination fractions in the order in which markers appear on the colLiv2 chromosome-level scaffolds. Overall, the marker order largely agrees with calculated recombination fractions; however, we identified a number of locations where pairwise recombination fractions suggest that portions of the colLiv2 chromosomes are not ordered properly, as exemplified in Figure 5. We also identified 42 markers for which the location with the best sequence match in colLiv2 appears to be incorrect based on recombination fraction estimates; these markers are summarized in Table S7 in File S1.

### Conclusions

The improved scaffold lengths and updated gene model annotations of Cliv_2.1 will further empower ongoing studies to identify genes responsible for phenotypic traits of interest. In addition, longer scaffolds will improve detection of regions under selection, including large deletions and other structural variants responsible for interesting traits in *C. livia*. Finally, our new transcriptomic data provide tissue-specific expression profiles for several adult tissue types and an important embryonic stage for the morphogenesis of limbs, craniofacial structures, skin, and other tissues.

## 

## Supplementary Material

Supplemental Material is available online at www.g3journal.org/lookup/suppl/doi:10.1534/g3.117.300443/-/DC1.
